# How does public opinion become extreme?

**DOI:** 10.1038/srep10032

**Published:** 2015-05-19

**Authors:** Marlon Ramos, Jia Shao, Saulo D. S. Reis, Celia Anteneodo, José S. Andrade, Shlomo Havlin, Hernán A. Makse

**Affiliations:** 1 Levich Institute and Physics Department, City College of New York, New York, NY 10031, USA; 2 Departamento de Física, PUC-Rio, 22451-900, Rio de Janeiro, Brazil; 3 CAPES - Coordenação de Aperfeiçoamento de Pessoal de Nível Superior, Ministério da Educação, 70040-020, Brasília, Distrito Federal, Brazil; 4 Bloomberg LP, New York, NY 10022, USA; 5 Departamento de Física, Universidade Federal do Ceará, 60451-970 Fortaleza, Ceará, Brazil; 6 Minerva Center and Physics Department, Bar-Ilan University, Ramat Gan 52900, Israel

## Abstract

We investigate the emergence of extreme opinion trends in society by employing statistical physics modeling and analysis on polls that inquire about a wide range of issues such as religion, economics, politics, abortion, extramarital sex, books, movies, and electoral vote. The surveys lay out a clear indicator of the rise of extreme views. The precursor is a nonlinear relation between the fraction of individuals holding a certain extreme view and the fraction of individuals that includes also moderates, e.g., in politics, those who are “very conservative” versus “moderate to very conservative” ones. We propose an activation model of opinion dynamics with interaction rules based on the existence of individual “stubbornness” that mimics empirical observations. According to our modeling, the onset of nonlinearity can be associated to an abrupt bootstrap-percolation transition with cascades of extreme views through society. Therefore, it represents an early-warning signal to forecast the transition from moderate to extreme views. Moreover, by means of a phase diagram we can classify societies according to the percolative regime they belong to, in terms of critical fractions of extremists and people’s ties.

The root causes of the rise of extreme opinions in society constitute nowadays a matter of intense debate among leading scholars^1^,^2^,^3^,^4^,^5^,^6^. Over the past few decades, there seems to be a worldwide trend towards the division of public opinions about several issues, e.g., political views, immigration, biotechnology applications, global warming, gun control, abortion, LGBT rights amongst many others. In many topics, a marked dwindling of moderate voices is found with the concomitant rising of extreme opinions^7^,^8^,^9^. Not only in politics but also in simple topics such as books, movies, fashion and other cultural topics, extreme positions sprout and the opinion or attitude of an initially small group could become the rule.

How these tendencies settle in society is still a mystery. The degree of social and economic development, the religious beliefs, the full history, and many other factors, undoubtedly, all contribute to mold the distribution of opinions of the members of a society. But, besides those social features contributing to a collective mood, interactions between individuals play also an important, often underestimated, role. In the social network defined by the ties between individuals, information, rumors, ideas, all travel. In this process, new opinions can take form and existing ones can be either strengthened or weakened. But to what extent does this interaction help to shape the public opinion? Can extreme views arise just from the interactions between individuals? The answers to these questions can help us to understand the dynamics of polarization of public opinions and make it possible to detect the trend to polarization.

Direct longitudinal statistics on the time evolution of individual opinions at the large scale are hard to obtain. Fortunately, large transverse data on the distribution of opinions of individuals about different particular issues are publicly available from surveys. These data have been obtained through polls, each one inquiring a broad sample of people about their attitude towards a specific subject, and offer a valuable evidence on the complex nature of public opinions. The responses are usually categorized into attitudes, e.g., very favorable, somewhat favorable, somewhat unfavorable and very unfavorable. For our purposes, people having very favorable or very unfavorable opinions can be defined as those holding (either positive or negative) extreme opinions.

The crux of the matter is to understand the dynamics of public opinion from the available transverse data in order to forecast the trend to polarization before it actually occurs^10^. From the analysis of these static data, we will extract clear evidence of radicalization in groups in the form of nonlinear behaviour near critical points and avalanche dynamics in belief spreading via critical transitions in the bootstrap percolation universality class. Such transitions shed light on the precise instance of the transition when groups adopt more extreme views.

The central finding of our paper is the discovery of a sharp statistical predictor of the rise of extreme opinion trends in society in terms of a nonlinear behaviour of the number of individuals holding a certain extreme view and the number of individuals with a moderate opinion and extreme opinion. We analyze polls embracing a wide range of issues such as religion, economics, politics, abortion, extramarital sex, the electoral vote, and opinion on everyday consumer products like books and movies. The surveys lay out a remarkable nonlinear predictor of the rise of extreme opinion views. This predictor is ubiquitous across the diversity of polls and surveyed countries, reflecting a remarkable generic feature of human opinion dynamics.

The nonlinear methodology signals a tipping point at which a society becomes extreme and has not been used before to predict opinion trends, as far as we know. The meaning of this nonlinearity is as follows. In general, for a statistical physics system of non-interacting agents, isometry is expected. This means that the system is extensive and the observables scale linearly with the system size^11^. That is, if we double the number of particles, the energy doubles as well, for instance. In terms of our social system of interest, a linear non-interactive extensive system implies that the number of extreme people should scale linearly with the number of people holding positive opinions. Thus, linearity is the byproduct of non-interactions among the agent. On the other hand, it is well known in statistical physics^11^, that correlations among the units, that appear specially near a phase transition, lead to nonlinear behaviour and non-extensivity. This effect is also called allometry in the field of socio-physics and is currently being investigated, for instance, in the scaling with the size of cities of different urban indicators like technology activity^12^ and health indicators^13^,^14^. For instance, it is found that the number of homicides scales superlinearly with the population of cities while the number of suicides scales sublinearly; both cases being examples of nonlinear allometric behaviour^12^,^13^.

The onset of nonlinear behaviour represents an early-warning signal forecasting an abrupt critical transition from moderate to extreme opinions, before it actually occurs. The nonlinear behaviour, which anticipates an abrupt change, is easily detectable in society via surveys and it measures the status of societies in the path towards predominance of extreme attitudes. By means of physical modeling, we find that the nonlinearity forecasts the onset of cascades of extreme view dissemination caused by the stubbornness of individuals. We show that the cascading is a consequence of an underlying bootstrap-percolation transition occurring at the tipping point when societies abruptly change from moderate to extreme.

## Results

### Empirical findings

To illustrate the polls, we consider a typical survey from the Pew Research Center (see Methods). Participants from a given country are asked whether they i) strongly believe, ii) believe, iii) disbelieve, or iv) strongly disbelieve that religion is an important part of their lives. Using these data, we first compute the fraction *f*_e_ of people holding an extreme view out of the total surveyed population in a given survey and country. That is, we compute *f*_*e*_ = *N*_*e*_^+^/*N* (or *N*_e_^−^/*N*), where *N*_*e*_^+^ (or *N*_*e*_^−^) is the number of people expressing an extreme positive view (or a negative one), and *N* is the total surveyed population. We then calculate the fraction of people holding moderate to extreme views: *f* = *N*^+^/*N* (or *N*^−^/*N*), where *N*^+^ (or *N*^−^) is the number of individuals believing and strongly believing in religion (or disbelieving and strongly disbelieving).

**Figure 1:**
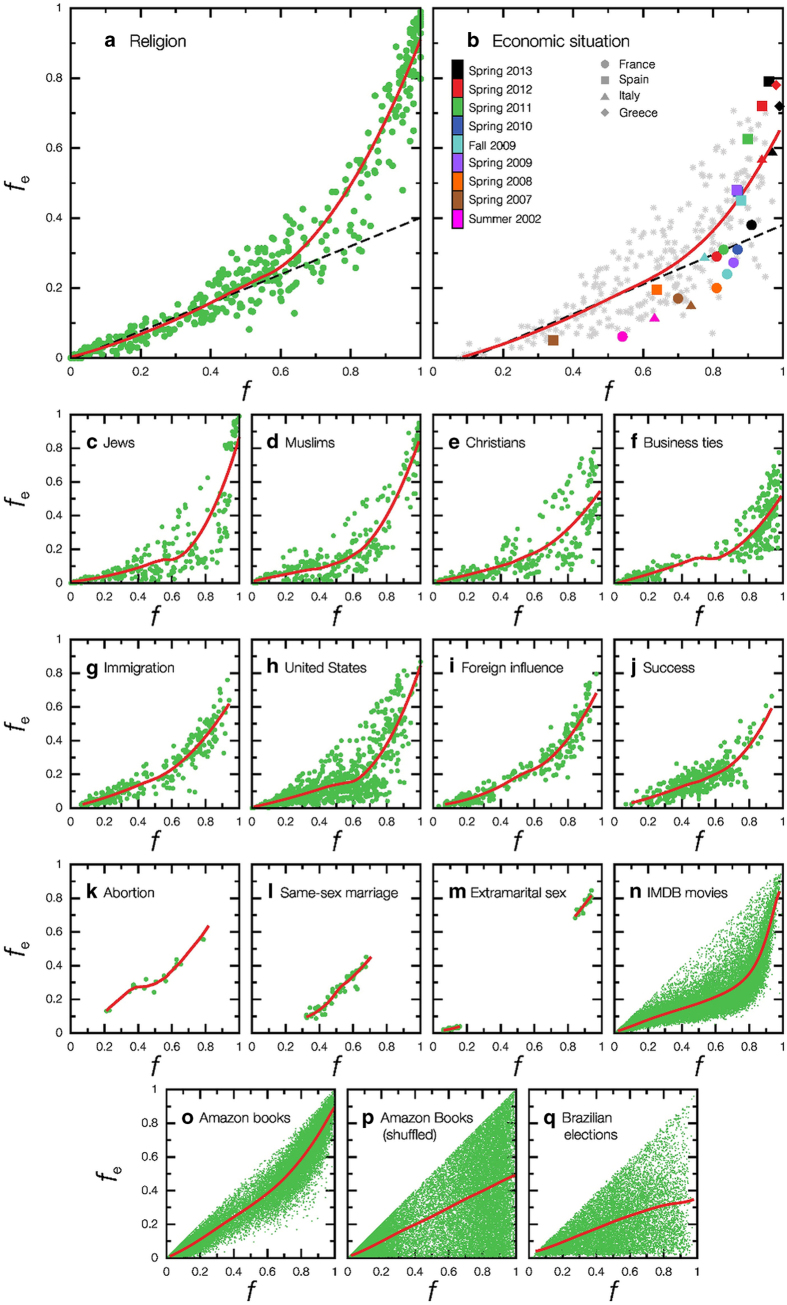
Empirical observations. Dependence of the fraction of extremists *f*_e_ on the fraction *f* of all people sharing an opinion, obtained from the outcomes of polls inquiring about a wide spectrum of issues as explained in Sec. Methods. For instance, in (**a**) participants from a given country are asked whether they *(i)* strongly believe, *(ii)* believe, *(iii)* disbelieve, or *(iv)* strongly disbelieve that religion is an important part of their lives. For each country and/or year, *f* and *f*_*e*_ were computed as explained in the text, for both favorable and unfavorable responses. The solid line is a nonparametric regression (see Methods). In (**a**) and (**b**) the dotted line depicts the linear behaviour expected for a non-interactive group. In (**b**) the fractions for unfavorable responses in surveys inquiring about the feeling on the economic situation are plotted. The time evolution (in color scale) of (*f*, *f*_*e*_) is depicted for France, Italy, Greece, and Spain. A nonlinear behaviour emerged in these countries after the European sovereign debt crisis of 2009.

Figure 1a displays *f*_*e*_ vs *f*, where each data point represents the result of the survey carried out in a given country and year. The set of points, although spread, are neatly correlated and follow a defined trend. To extract the main relationship between *f*_*e*_ and *f* without predetermined functional form, we use nonlocal regression LOESS^15^ with span *h* = 0.8 as well as the Nadaraya-Watson estimator^16^,^17^ (see Sec. Methods for details). The regression is represented by the solid line in Fig. 1a. The result is paradigmatic of the nontrivial dependency of *fe* on *f* that defines the early-warning signal at which a society starts to become extreme. For a relatively small fraction of extremists, *f*_*e*_ is approximately proportional to *f* (dotted straight line in Fig. 1a). This linear behaviour can be interpreted as arising from a system of non-interacting individuals who form their opinions independently from each other. In the absence of interactions among people, the linear regime would extend up to *f* = 1. However, at *f*_*e*_ ≈ 0.20, a noticeable departure from linearity is observed. A nonlinear behaviour ensues, marking the onset of a surplus of extremists in comparison with the expected number in the linear (non-interactive and extensive) case. Therefore, when a significant majority is reached in a given population, the interaction between individuals with similar opinion leads to a shift from moderate opinion to the extremism. This phenomenon is in good agreement with the scenario reported for the case of very small groups in behavioural sciences^18^.

A typical case study of transition towards extreme views is the opinion about the economic situation after the European sovereign debt crisis of 2009. The time evolution of (*f*, *f*_*e*_) for France, Italy, Greece, and Spain in Fig. 1b shows that nonlinear behaviour emerges after 2009, indicating that extreme negativism has prevailed across societies. This result supports the hypothesis that the departure from linearity marks the rise of extreme views.

The observed nonlinearity is not a prerogative of religious or economic issues where opinions frequently appear to be polarized, but extends to many kinds of polls across the globe. Polls ranging from abortion to immigration (see details in Methods) are presented in Fig. 1, all displaying similar features. It is a surprisingly ubiquitous behaviour also found on much simpler issues such as opinions on books and movies (Fig. 1n–o). Although the precise shape of (*f*, *f*_*e*_)-curves changes from one poll to another, there seems to be a universal trend very different to that found, for instance, in shuffled data (Fig. 1p).

In Fig. 1q, we show the results for state deputies, each point corresponds to one city for which we compute the fractions of votes within each political orientation, as done for the other polls. This is a remarkable counter-example. We find a dispersion pattern in (*f*, *f*_*e*_) similar to that which would appear if people had chosen the political orientation of the candidates (from extreme left to extreme right) in a random fashion. Indeed, the voting data appear to be uncorrelated in similar way as that obtained in the randomized data on books, Fig. 1p. Further research is needed to reveal whether the absence of a trend in the Brazilian electoral vote is a generic feature of elections at large.

In what follows we interpret the nonlinear behaviour in terms of an underlying critical transition from moderate to extreme views taking place in society. Remarkably, the departure from linear behaviour, which appears for moderate *f*_e_, forecasts a critical point marking the precise transition from moderate behaviour to extreme views. Consequently, the (*f*, *f*_*e*_)-curve, which can be easily obtained from polls, readily predicts the onset of extreme opinion before the actual transition has been materialized.

### Modeling extreme opinion dynamics

The features observed here cannot be explained by existing opinion models, as far as we know. Most of them lead to consensus of a single opinion or to equal fractions of opinions^19^,^20^,^21^. Other ones allow coexistence of minority and majority opinions^22^,^23^,^24^,^25^, but are not suitable to describe the empirical data where we need to distinguish extreme from moderate opinions. All these models may constitute a sufficient simplification to tackle certain problems, but they are not suitable to study the emergence of extremisms where we need to distinguish extreme from moderate opinions. There are also the so-called *bounded confidence* models^2^,^26^,^27^ that assume that only people with sufficiently close attitudes interact. These models have been considered to study extreme opinion dynamics, but lead to discontinuous distributions of opinions.

These observations call for a comprehensive simple model to capture the underlying microscopic origin of extreme opinion formation. We propose a network model where the opinion of an individual, *q*, takes real values between −1 and +1. Extreme opinion is considered for |*q*|>*q*_*e*_ and positive (negative) opinion starts for *q* > 0 (*q* < 0). Motivated by the four questions of most polls, one can consider *q*_*e*_ = 0.50.

**Figure 2:**
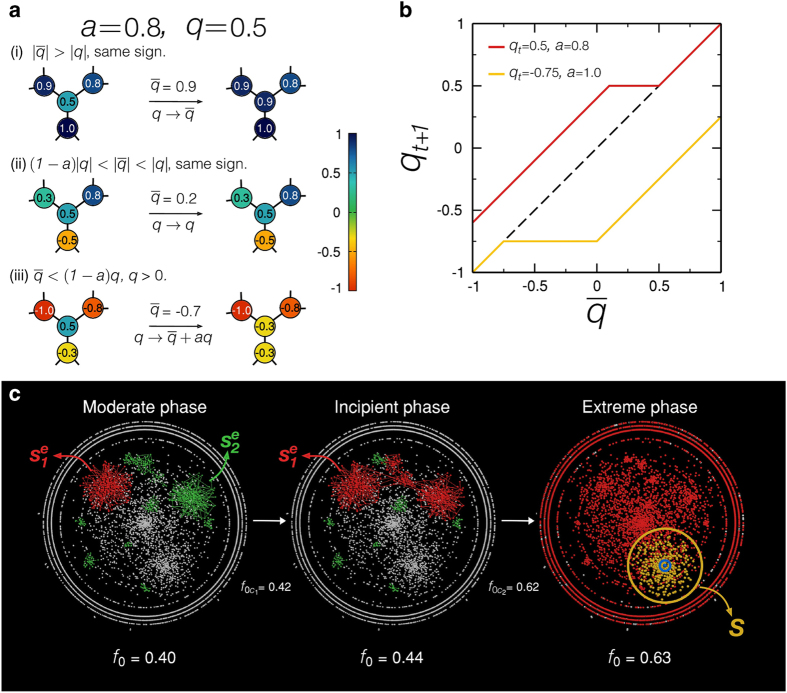
Opinion model. (**a**) Consider a node with degree 3 which holds a moderate opinion *q* = 0.5, and stubbornness *a* = 0.8. There are three possible situations in the model rules according to 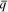: *(i)* If 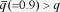 and of the same sign as *q*, the node’s opinion becomes more extreme, 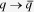. *(ii)* If 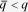 but larger than a fraction of *q* given by 1 − *a*, then the nearest neighbors cannot change the opinion of the node due to its stubbornness. Consequently, the opinion remains the same. *(iii)* When the average opinion of the nearest neighbors is more moderate or opposite in sign (as in the panel), it can influence the node’s opinion. Since in this case 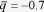, the positive opinion of the node changes, becoming *q* = −0.3. (**b**) Diagram showing the new opinion of a node at step *t* + 1, *q*_t+1_, as a function of the average opinion of the node’s nearest neighbors at step *t*, 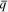. Two typical cases are depicted. Red curve: with moderate positive opinion *q*_*t*_ = 0.5 (blue) and moderate stubbornness *a* = 0.8 (this case corresponds to (**a**)) Orange curve: with extreme negative opinion *q*_*t*_ = −0.75 and stubbornness *a* = 1. The larger the value of *a* the wider the inflexibility range of rule *(ii)* where opinion does not change. (**c**) Illustration of the different phases for different values of *f*_0_ in a typical ER network of size *N* = 10000 and 〈*k*〉 = 4. In Moderate Phase I, extremist clusters are mostly isolated. The largest e-cluster is in red and top ten in green, white nodes are moderate, most of the activity is concentrated in the 3-core, and the concentric circles are the 1 and 2-shells 39,40,41. In Incipient Phase II an incipient giant e-cluster first appears (red). The system is increasingly more susceptible to perturbations; the yellow cluster in Extreme Phase III depicts a cascade resulting by the change to extreme of a single node in blue. Deep in the Extreme Phase III most nodes (in red) have become extremists.

We introduce a parameter *a* (0 ≤ *a* ≤1) which gauges the stubbornness of individuals, a realistic ingredient that we show to be crucial to understand the nonlinear behaviour in opinion spreading. The dynamics considers the previous opinion of the individual as well as the average opinion 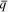 of the neighbors in the network according to (see also Fig. 2):

*(i)* 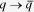, if 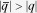 and *q* has the same sign as 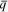.

*(ii) q → q*, if 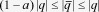 and *q* has the same sign as 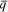.

*(iii)* 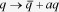, if [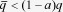 and *q* > 0] or [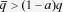 and *q* < 0].

Rule *(i)* determines that a node will adopt the average opinion of its neighbors if this average is more extreme that the node’s opinion. In fact, it is sound that people with a weak opinion will be more likely influenced by people with a stronger one. Notice that, even if the stubbornness parameter does not participate explicitly in this rule, a subject who has a stronger opinion than its contacts results to be more inflexible, since it is more difficult to change its opinion. According to rule *(ii)*, no changes occur for a range of intermediate opinions, this range being wider the larger the stubbornness and the more stronger the node’s opinion. Finally, rule *(iii)* determines that, when the average opinion of the neighbors is either opposite to or much less extreme than the node’s opinion, then the new opinion is 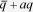. That is, the new opinion is determined not only by friends, but also partially by its own opinion, weighted by the stubbornness *a*. Thus, the role of stubbornness *a* is twofold: if *a* is large, not only 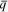 should be farther enough from *q* in order to change the node’s opinion, but *a* also reduces the relative effect of its neighborhood. In the limiting case *a* = 0, the inflexibility range collapses mimicking the most flexible individuals, easily influenced by the close environment and assuming the average value of the neighbors, similarly to majority rule models^20^,^21^.

Stubbornness is a crucial ingredient to have an heterogeneous population with different opinions. In the absence of stubbornness (*a* = 0), all the three rules reduce to the single prescription of adopting the average value of the neighbors, yielding consensus of a single opinion as in the majority rule model of Refs. 20, 21. Differently, when setting *a* > 0, people with initially different opinions will not be easily convinced and heterogeneity of opinions will persist in the final state, yielding a continuous probability density function of opinions. Note that here stubbornness is conceptually different from previous definition using local fields^28^, or from the idea of intransigent individuals, where inflexible nodes do not change their opinion^29^,^30^.

We simulate the model on an Erdös-Rényi (ER) network, a general class of random networks with a Poisson degree distribution and with the small-world property^31^, with average degree 〈*k*〉, starting with *f*_0_ fraction of nodes with positive opinion. To define the initial state of the opinion dynamics on top of a chosen network of size *N*, we select *f*_0_ that gives the initial fraction of nodes with positive opinion. After that, we select *f*_0_*N* nodes and assign to each one of them a random opinion value *q* uniformly distributed between 0 and +1. To the remaining (1 − *f*_0_)*N* nodes, we assign a random value of *q* uniformly distributed between −1 and 0. Then, at each time step *t* the opinions *q* of all nodes in the network are synchronously updated according to the rules defined above. Positive extremists are a minority for any initial condition. We then compute the fractions *f* and *f*_*e*_ in the final state controlled by *f*_0_.

**Figure 3:**
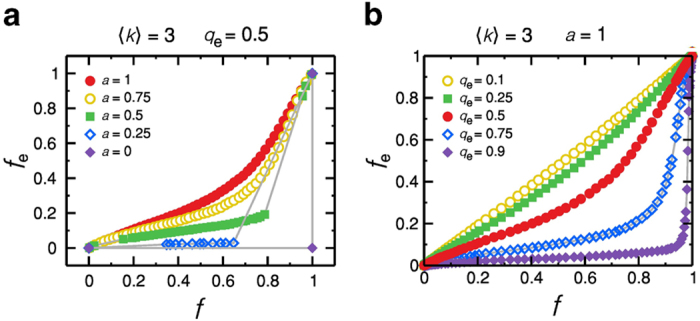
The effect of *a* and *q*_*e*_. (**a**) Fraction of extremists *f*_*e*_ as a function of *f* for different values of *a* and *q*_*e*_ = 0.5. When we lower the values of the stubbornness *a* the model is reduced to the consensus model. (**b**) When we vary *q*_*e*_ for a fixed *a*, the behaviour of *f*_*e*_ as a function of *f* exhibits non-realistic profiles for *q*_*e*_ close to 0 or 1. For *q*_*e*_ close to 0, a one-to-one relation between *f*_*e*_ and *f* is found. When *q*_*e*_ is close to the 1, a majority of extremists emerges when *f* → 1. Both regimes contradict the empirical findings, therefore the choice of intermediate values of *q*_*e*_ is more realistic.

**Figure 4:**
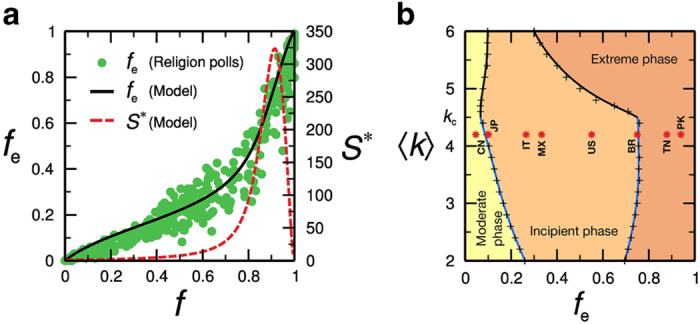
Model and poll data. (**a**) *f*_*e*_ vs *f* for the religion polls and fitting using the model with N = 10^4^, *a* = 1 and 〈*k*〉 = 4.2. The model closely matches the empirical result. We plot the largest avalanche size S* obtained by damaging the network as explained in the text. The onset of nonlinear behaviour and cascading avalanches coincide. (**b**) Extreme phase diagram from modeling in terms of *f*_*e*_ and 〈*k*〉. The transition lines separating the three phases at 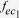 and 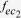 are analogous to 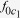 and 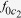, respectively. Black lines correspond to first-order transitions for 〈*k*〉 > *k*_c_, and blue lines correspond to continuous transitions for 〈*k*〉 > *k*_c_. Moderate Phase I: there is no giant e-cluster. Incipient Phase II: a giant e-cluster appears, with increasing cascading effects. Extreme Phase III: characterized by the consensus of extremists for sufficiently high mean degree. The symbols represent selected countries from religion polls in (**a**) encompassing the whole spectrum of phases (names in Internet two-letter code). 〈*k*〉 should be interpreted as the effective average degree through which opinion spreads rather than the actual number of ties of the individuals which could be much larger. The effective average degree is obtained from the fitting in (**a**).

As shown in Fig. 3, the model presents a rich variety of behaviours for the dependence of *f*_*e*_ with *f* Fig. 3a shows the reduction of the model into the consensus model for fixed values of 〈*k*〉 and *q*_*e*_. When *a* is large enough (*a* = 1 and 0.75), one can find a continuous spectrum of *f*_*e*_ as a function of *f*, ranging from *f* = 0 to *f* = 1. When we lower *a*, a discontinuity emerges, and the fraction *f*_*e*_ collapses and becomes dominant only for large values of *f*. For *a* = 0, our model reaches the consensus regime and every node adopts a single opinion given by the average of the initial distribution of *q*. In this regime, considering that we set an uniform distribution of *q*, if *f*_*0*_ < 0.5, *f* and *f*_*e*_ are null. However, when *f*_0_ > 0.5 the positive values of *q* takeover, and *f* = 1. As depicted in Fig. 3a, for *a* = 0 and *f* = 1, *f*_*e*_ assumes two values. In this case, *f* = 0 if the average of the initial distribution of *q* is lower than *q*_e_ = 0.5, and *f* = 1 if the average of the initial distribution of *q* is larger than *q*_*e*_ = 0.5. Figure 3b presents how the model behaves by varying *q*_*e*_ for fixed 〈*k*〉 and *a*. For low *q*_*e*_, for instance *q*_*e*_ = 0.1, one can notice an one-to-one linear relation between *f* and *f*_*e*_, once almost all positive *q* are classified as extreme. Increasing *q*_*e*_ diminishes the slope of the linear portion of low *f*_*e*_, and for high *q*_*e*_ a sudden and non-realistic emergence of extremism is found. Therefore, based on the empirical results presented in Fig. 1, and since we are interested in non-consensus regimes, we consider without losing generality the representative set of parameters *a* = 1 and *q*_*e*_ = 0.5. As shown in Fig. 4a, the model with *a* = 1, *q*_*e*_ = 0.5, and 〈*k*〉 = 4.2 mimics very well (*f*, *f*_*e*_) of religion data.

### Phases of extreme opinion

Next, we discuss how the phenomenology of the model allows us to interpret the nonlinearity in terms of changes in the microscopic dynamics of beliefs spreading. These changes are expressed in well-defined transitions between the different phases of the final state depicted in Fig. 2c. The transitions from one phase to another are characterized by the percolative behaviour of extremists and their networks of contacts. The behaviour of the connected components of extremists (named *e-clusters*, Fig. 2c) reveals the origin of the nonlinearity. Changing *f*_0_, the system passes through three distinct phases separated by two critical transition points as exemplified in Fig. 2c. The phenomenology of the transitions is closely related to activation models like bootstrap percolation^32^,^33^,^34^,^35^, the opinion model of Watts^4^,^36^ and the multi-percolation model of competition of innovations of Helbing *et al.*^37^. Indeed, there is a correspondence between the dynamics of vertex activation in bootstrap percolation^32^,^34^ and the change from moderate to extreme opinions (*e-activation*) in our model, both start from an initial configuration where nodes are active with probability *f*_e_^*0*^ = (1 − *q*_*e*_)*f*_0_ (see below).

The purpose of the model is then to interpret the nonlinear behaviour in terms of critical phase transitions which cannot be directly measured from real data since the contact network of ties is usually unknown at the large scale. The model identifies the following phases:

#### Moderate Phase I

**Figure 5:**
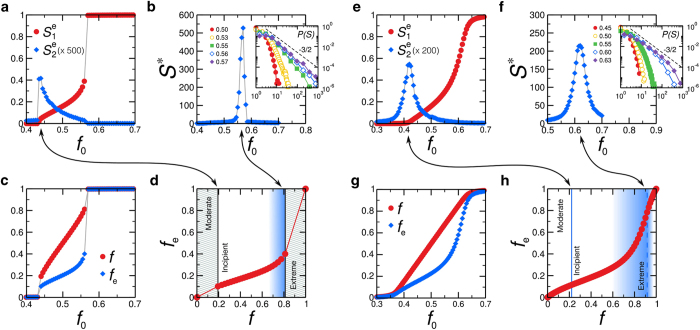
Critical transitions. (**a**)-(**d**) For 〈*k*〉 = 5 > *k*_*c*_. (**e**)-(**h**) For 〈*k*〉 = 4 < *k*_*c*_. Displayed results are an average over 50 ER networks (except for (**b**) and (**f**) where we use 300 networks) and we set *a* = 1. (**a**) and (**e**) 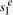 and 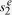 vs *f*_0_. Cluster sizes are normalized by the size of the largest component of the network (*N* = 10^5^). (**b**) and (**f**) Largest cascade size S* vs *f*_0_. The inset shows the distribution of cascade sizes for different values of *f*_0_, exhibiting power-law scaling (*N* = 10^4^). (**c**) and (**g**) *f*_*e*_ and *f* vs *f*_0_ (*N* = 10^5^). (**d**) and (**h**) Curves *f*, *f*_*e*_ to highlight the nonlinear behaviour. The hatched regions in (**d**) correspond to the jumps in the first-order transitions, hence inaccessible in the infinite size limit. The bluish colored areas in (**d**) and (**h**) represent the region of large cascading S* regime from (**b**) and (**f**), respectively. They show that the nonlinearity is associated with the occurrence of progressively larger cascades as *f* increases. For clearness, we consider only the fraction of positive vertices (*q* > 0) and extreme positive ones (*q*_e_ > 0) in the calculation of *f* and *f*_*e*_ .

For low *f*_0_, we observe small isolated e-clusters. The size of the largest e-cluster, 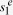, as a function of *f*_0_ vanishes (Fig. 5) and the behaviour of (*f*, *f*_*e*_) remains approximately linear.

#### Incipient Phase II

Above a critical value, 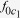, a giant e-component of size 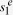 emerges which occupies a non-vanishing fraction of the network (Figs. 5a and 5e). The critical point 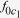 is also signaled by the peak in the size of the second largest e-cluster, 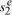. The order of this transition is determined by 〈*k*〉 in comparison with a critical value *k*_c_ = 4.5 ± 0.1. For 〈*k*〉 > *k*_*c*_, 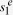 (Fig. 5a), as well as *f*_*e*_ and *f* (Fig. 5c), present a discontinuity at 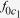; a fingerprint of an abrupt first-order transition. For 〈*k*〉 < *k*_*c*_, the transition is second order like in ordinary percolation. The size 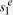 increases continuously at 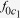, 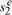 presents a peak (Fig. 5e), and *f*_*e*_ and *f* also increase smoothly (Fig. 5g).

After a giant e-cluster appears, a collective phenomenon in avalanches of extreme opinion spreading takes place. We quantify the avalanche dynamics inspired by similar dynamics appearing in bootstrap percolation^34^,^35^.

In bootstrap percolation^34^,^35^ nodes in a given network can take two values, active or inactive. At the beginning of the dynamics, a fraction *f*_*a*_ of nodes chosen at random are set into the active state, the rest are inactive. An inactive node becomes active only if it has at least *k* active neighbors, where *k* is a fixed parameter of the model, while active nodes remain in this state forever. The activation rule is iteratively applied until the system reaches a final state with no further changes. A variant has been introduced by Watts^4^ in which the activation condition is given by a minimal fraction of active neighbors, instead of a minimal fixed number of neighbors *k*.

In bootstrap percolation, when a giant cluster of active nodes exists, an infinitesimal change of the fraction of active nodes can trigger an avalanche of activations. This cascade process is related to the existence of sub-critical clusters of activatable nodes. A node belongs to a subcritical cluster if its number of active neighbors external to the cluster is one less that the threshold degree necessary for activation^34^. When a sub-critical node gains an active neighbor, it becomes active and, as a consequence, its connected neighbors in the cluster in turn gain a new active neighbor, and a cascade occurs. In contrast, in our case, the activation rules are far more complex to allow a clear definition of sub-critical nodes. In fact, the e-activation itself of a vulnerable node does not guarantee the activation of its activatable nearest neighbors. Furthermore, indirect activation is also possible: a node *i* might be transitively activated through some already activated intermediary, as soon as the node *i* receives an extra contribution to its 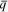 due to the modification of one of its nearest neighbors.

In order to detect and characterize the possible avalanches, we circumvented that difficulty by perturbing the system. We choose a node with opinion 0 < *q* < *q*_*e*_ = 0.5 and substitute it by *q* = 1, measuring the number of vulnerable nodes *S* that become extremist in the new stable state. Figure 2c shows the result of the process described above.

We accumulate data for all nodes with opinions below *q*_*e*_ (triggered one at a time) that succeeded in triggering an avalanche and repeat for several realizations. The average size of the avalanches 〈*S*〉 and the largest avalanche size S* were computed as a function of *f*_0_. We find that *S* is small around 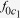 but increases rapidly with *f*_0_. The largest avalanche size S* as a function of *f* is plotted in Fig. 4a. It indicates that the nonlinear trend in (*f, f*_*e*_) in model and empirical data is accompanied by the increase of avalanche sizes. Thus, we associate the onset of the nonlinear regime with the incipient extreme phase where the system starts to be susceptible to changes, and small perturbations can generate a cascade of extreme opinion spreading.

#### Extreme Phase III

**Figure 6:**
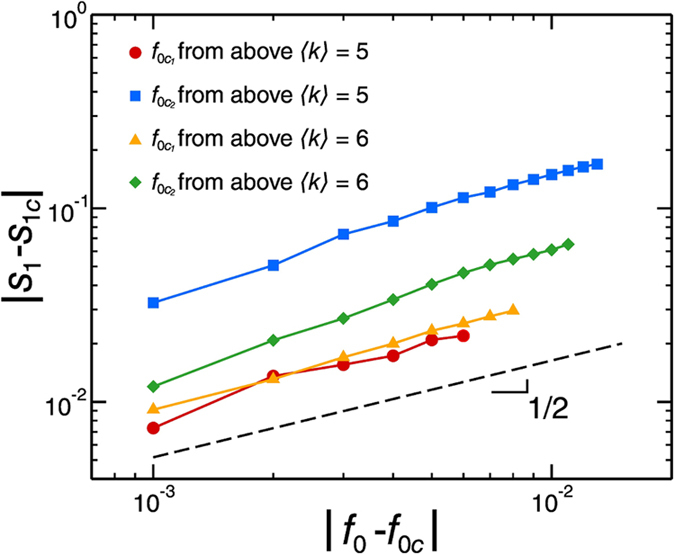
Scaling at the hybrid transition, for two cases of high connectivity: 〈*k*〉 = 5 and 〈*k*〉 = 6, at the two critical points. The exponent is close to 1/2, that of bootstrap percolation. We used ER networks of size 10^5^.

S* peaks at a second transition point 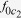 (Fig. 5b and 5f) signaling the transition to a phase where the whole society has become extreme. This transition can be smooth or abrupt according to 〈*k*〉. If 〈*k*〉 > *k*_c_, the transition is sharp and first-order. The distribution of avalanche size develops a power-law tail with scaling exponent 3/2 (inset Fig. 5b). The value of this critical exponent suggests that the model is in the universality class of bootstrap percolation^34^,^35^,^37^. Furthermore, the activation dynamics in bootstrap percolation^34^ and the opinion model of Watts^4^ exhibit hybrid transitions as in our model: a combination of a jump (as in first order transitions) and a power law (as in second order transitions) near the critical point. Close to the critical point, the size of the largest e-cluster behaves like
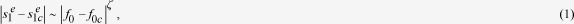
where 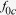 refers to either 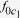 and 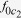, and with the exponent ζ ≈ 1/2 (see Fig. 6), like in bootstrap percolation^34^,^35^. We notice that these are hybrid transitions, and the approach to the critical point in terms of power laws is given from above and below for 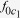 and 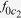, respectively. This result further suggests that our model, although not the same as bootstrap percolation, could be in the same universality class.

The sharp peak of S* (Fig. 5b) reflects the discontinuity in 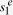 at 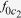 (Fig. 5a), which is also seen in *f* and *f*_*e*_ (Fig. 5c). After this abrupt jump, almost all nodes belong to the giant e-cluster. When 〈*k*〉 > *k*_c_, S* presents a smeared peak at 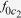 (Fig. 5f). The 3/2 power-law decay found for 〈*k*〉 > *k*_c_ applies approximately to the envelope of the distributions of avalanche sizes (inset Fig. 5f). In this case, the approach to the extreme phase is progressive in terms of *f,* and *f*_*e*_ (Fig. 5g,h).

The impact of this critical scenario on (*f, f*_*e*_) is illustrated in Figs. 4a, 5d and 5h. They show that the onset of nonlinearity in the Incipient Phase II is associated to the increase of cascade sizes. The origin of nonlinearity is the presence of cascades of extremists in phase II and the onset of nonlinearity is a predictor of more drastic changes that occur when the size of the avalanches becomes maximal.

The different phases predicted by the model are represented in Fig. 4b into a phase diagram defined in terms of precise critical values of *f*_*e*_ and 〈*k*〉. It displays the line of percolation transition separating moderate and incipient extreme phases predicted by the model, whose main trait is the absence and presence of a giant e-cluster, respectively, and the transition to the extreme phase. In the case of religion polls, we find 〈*k*〉 = 4.2 in Fig. 4b which is obtained by fitting the data (*f, f*_*e*_) in Fig. 4a using all the data points from all the countries. Once the value of 〈*k*〉 is obtained, then we can plot the particular countries in the phase diagram since we also know exactly the value of *f*_*e*_.

By means of the interpretation provided by the model, we classify societies according to their extreme level; the phase diagram measures the status of societies in the path towards predominance of extreme attitudes. Selected data from religion polls from Fig. 4a are projected onto the phase diagram, Fig. 4b. Most of the countries are located in Phase II and a few are in Phase III, where the majority of the population has become extreme. For instance, we find that, in terms of positive opinion about how religion is important in peoples life, Pakistan and Tunisia are in Phase III, while Brazil is at the transition point between Phase II and III. USA is also very close to the transition point closely approaching Phase III. We notice that the position of a country in the phase diagram can be changed by an increase or decrease of either *f*_*e*_ or, more importantly, 〈*k*〉. The effective degree can be easily increased by the use of social media, for example. Thus, for instance, USA might enter Phase III in religion attitude by just increasing its effective degree from its current 〈*k*〉 = 4.2 to 〈*k*〉 = 5. This would produce a first order abrupt transition to Phase III. Other countries like Mexico, Italy and Japan are in the incipient Phase II. Finally, China belongs to the moderate Phase I in terms of positive attitudes towards religion.

This classification may have important implications, since we could detect whether a country is at the edge of an abrupt change to extreme phase produced either by an increase of *f*_*e*_ or 〈*k*〉 (for instance, by increasing connectivity by the use of social media).

## Conclusions

A natural situation for extreme behaviour is human opinion as studied here. The consistency between real data and model predictions is suggestive of a possible broader scope of the present statistical analysis. This good agreement makes it a candidate for predictor of other aspects of human collective behaviour involving beliefs and decision-making where opinion cascades prevail^4^, such as competition of market innovations^37^,^38^. For instance, the nonlinear early-signature might be able to anticipate wide adoption of consumer products, as soon as the nonlinearity appears in consumer ratings of items such as books and movies. Further research is planned to investigate the applicability of nonlinear analysis to human collective behaviour at large.

## Methods

### Nonparametric regression

We consider nonparametric regression procedures to obtain a smooth set of points from each set of scattered data (*x*_*i*_, *y*_*i*_), *i* = 1, ... ,*n*, as those in Fig. 1: the locally weighted regression (LOESS) and the Nadaraya-Watson (NW) regression.

We used LOESS, with span *h* = 0.8 to extract the main trend of (*f*, *f*_*e*_) as well as the NW estimator.

*LOESS:* the estimated values 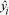 for each point *x*_*i*_ are obtained through a weighted least-squares fitting procedure 15. A weight function *W* that depends on the distance *h*_*i*_ to the *r*th nearest neighbor of point *i* is used. The *k* = 1, … ,*n*, (with *k* ≠ *i*) weights for each point *x*_*i*_ are given by
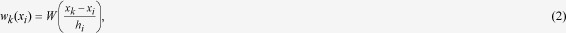
where *W* is the tricubic weight function
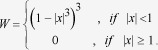

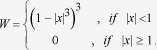


Equation (2) determines the estimated 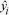 in reference^15^.

*Nadaraya-Watson:* we construct the kernel smoother function^16^.
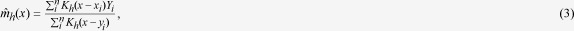
where *K*_*h*_(*x* − *x*_*i*_) is a Gaussian kernel of the form,
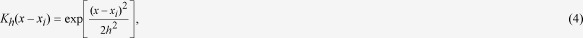


with bandwidth *h* estimated by least squares cross-validation method.

### Description of polls used in Fig. 1

We provide information about the data used in each panel depicted in Fig. 1. For the survey data, obtained for example from the Pew Research Center, we present explicitly, when available, *(i)* the question used in each survey, *(ii)* the original URL where the data can be found, *(iii)* the number of countries where the surveys were performed, *(iv)* the number of surveys performed which is larger than the number of countries in *(iii)* since the surveys are performed over many years for a given country, and *(v)* the dates when the surveys were performed.

- Religion:Question: How important is religion in your life -- very important, somewhat important, not too important, or not at all important?Source: Pew Research CenterURL: http://www.pewglobal.org/question-search/?qid=408&cntIDs=&stdIDs=Total number of countries: 59Total number of surveys: 231Surveys date: Summer 2002, Spring 2005, Spring 2006, Spring 2007, Spring 2008, Spring 2009, Fall 2009, Spring 2010, Spring 2011, Late Spring 2011, and Spring 2012.
- Economic situation:Question: Now thinking about our economic situation, how would you describe the current economic situation in (survey country) - is it very good, somewhat good, somewhat bad or very bad?Source: Pew Research CenterURL: http://www.pewglobal.org/question-search/?qid=753&cntIDs=&stdIDs=Total number of countries: 59Total number of surveys: 260Surveys date: Summer 2002, Spring 2007, Spring 2008, Spring 2009, Fall 2009, Spring 2010, Spring 2011, Late Spring 2011, Spring 2012, and Spring 2013.The time evolution is shown for the following cases:Countries: France, Italy, Greece, and Spain.Total number of surveys: 24Surveys date: Summer 2002 (France and Italy), Spring 2007 (France, Italy, and Spain), Spring 2008 (France and Spain), Fall 2009 (France, Italy, and Spain), Spring 2009 (France and Spain), Spring 2010 (France and Spain), Spring 2011 (France and Spain), Spring 2012 (France, Italy, Greece, and Spain), and Spring 2013 (France, Italy, Greece, and Spain).
- Jews:Question: Please tell me if you have a very favorable, somewhat favorable, somewhat unfavorable, or very unfavorable opinion of JewsSource: Pew Research CenterURL: http://www.pewglobal.org/question-search/?qid=834&cntIDs=&stdIDs=Total number of countries: 32Total number of surveys: 131Surveys date: Spring 2004, Spring 2005, Spring 2006, Spring 2008, Spring 2009, Spring 2010, Spring 2011, and Late Spring 2011.
- Muslims:Question: Please tell me if you have a very favorable, somewhat favorable, somewhat unfavorable, or very unfavorable opinion of MuslimsSource: Pew Research CenterURL: http://www.pewglobal.org/question-search/?qid=836&cntIDs=&stdIDs=Total number of countries: 32Total number of surveys: 135Surveys date: Spring 2004, Spring 2005, Spring 2006, Spring 2008, Spring 2009, Spring 2010, Spring 2011, and Late Spring 2011.
- Christians:Question: Please tell me if you have a very favorable, somewhat favorable, somewhat unfavorable, or very unfavorable opinion of ChristiansSource: Pew Research CenterURL: http://www.pewglobal.org/question-search/?qid=828&cntIDs=&stdIDs=Total number of countries: 32Total number of surveys: 133Surveys date: Spring 2004, Spring 2005, Spring 2006, Spring 2008, Spring 2009, Spring 2010, Spring 2011, and Late Spring 2011.
- Business ties:Question: What do you think about the growing trade and business ties between (survey country) and other countries - do you think it is a very good thing, somewhat good, somewhat bad or a very bad thing for our country?Source: Pew Research CenterURL: http://www.pewglobal.org/question-search/?qid=1011&cntIDs=&stdIDs=Total number of countries: 55Total number of surveys: 184Surveys date: Summer 2002, Spring 2007, Spring 2008, Spring 2009, Spring 2010, Spring 2011, and Late Spring 2011.
- Immigration:Question: As I read another list of statements, for each one, please tell me whether you completely agree, mostly agree, mostly disagree or completely disagree with it… We should restrict and control entry of people into our country more than we do now.Source: Pew Research CenterURL: http://www.pewglobal.org/question-search/?qid=54&cntIDs=&stdIDs=Total number of countries: 54Total number of surveys: 128Surveys date: Summer 2002, Spring 2007, Spring 2009, and Fall 2009.
- United States:Please tell me if you have a very favorable, somewhat favorable, somewhat unfavorable, or very unfavorable opinion of the United States.Source: Pew Research CenterURL: http://www.pewglobal.org/question-search/?qid=844&cntIDs=&stdIDs=Total number of countries: 59Total number of surveys: 351Surveys date: Summer 2002, March 2003, May 2003, Spring 2004, Spring 2005, Spring 2006, Spring 2007, Spring 2008, Spring 2009, Spring 2010, Spring 2011, Late Spring 2011, Spring 2012, and Spring 2013.
- Foreign influence (protection against):Question: As I read another list of statements, for each one, please tell me whether you completely agree, mostly agree, mostly disagree or completely disagree with it… Our way of life needs to be protected against foreign influence.Source: Pew Research CenterURL: http://www.pewglobal.org/question-search/?qid=51&cntIDs=&stdIDs=Total number of countries: 52Total number of surveys: 119Surveys date: Summer 2002, Spring 2006, Spring 2007, Spring 2009, and Spring 2012.
- Success (determined by external forces):Question: Please tell me whether you completely agree, mostly agree, mostly disagree or completely disagree with the following statement… Success in life is pretty much determined by forces outside our control.Source: Pew Research CenterURL: http://www.pewglobal.org/question-search/?qid=908&cntIDs=&stdIDs=Total number of countries: 55Total number of surveys: 155Surveys date: Summer 2002, Spring 2007, Spring 2008, Spring 2009, Fall 2009, Spring 2011, and Late Spring 2011.
- Abortion:Question: Do you agree very much, a little, not really, not at all with the statement… If a woman doesn’t want children, she should be able to have an abortion.Source: Euro RSCG/TNS SofresURL: http://en.wikipedia.org/wiki/Societal_attitudes_towards_abortionTotal number of countries: 10 (European only)Total number of surveys: 10Surveys date: May 2005
- Same-sex marriage:Question: Please tell me whether you strongly favor, favor, oppose, or strongly oppose it… Allowing gay and lesbian couples to marry legally?Source: Pew Research CenterURL:  http://www.pewglobal.org/question-search/?qid=828&cntIDs=&stdIDs=Total number of countries: 1Total number of surveys: 28Surveys date: May 1996-October 2012
- Extramarital sex:Question: What about a married person having sexual relations with someone other than the marriage partner, it is always wrong, almost always wrong, wrong only sometimes, or not wrong at all?Source: NORC/GSSURL:  http://pt.scribd.com/doc/131666438/Polls-on-Attitudes-on-Homosexuality-Gay-MarriageTotal number of countries: 1 (USA)Total number of surveys: 23Surveys date: 1973-2010
- IMDB Movies:We collect ratings (from 1 to 10 stars) of imdb.com movies with number of opinionators greater than 1,000. We crawled all the votes until March 28, 2013. We exclude TV episodes. Each datapoint in Fig. 1n is a movie out of the 19,405 total. We convert the star ratings into opinion as follows:extreme positive opinion (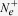): 9 and 10 stars,positive opinion (N^+^): 7, 8, 9 and 10 stars,negative opinion (*N*^−^): 1, 2, 3 and 4 stars,extreme negative opinion (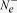): 1 and 2 stars.URL: http://www.imdb.com/search/title?at=0&sort=release_date_us&title_type=feature,tv_movie,tv_series,tv_special,mini_series,documentary,game,short,video,unknown&user_rating=1.0,10Total number of movies: 301,743; with more than 1,000 ratings: 19,405
- Amazon Books:We collected ratings, from 1 to 5 stars, of books at sale on amazon.com with a minimum of 50 opinionators. Each datapoint in Fig. 1o is a book out of the total of 16,390. We convert the star ratings into opinion as follows:extreme positive opinion (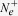): 5 stars,positive opinion (*N*^+^): 4 and 5 stars,negative opinion (*N*^−^): 1 and 2 stars,extreme negative opinion (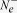): 1 star.URL: http://www.amazon.com/Total number of books: 291,428; with more than 50 ratings: 16,390
- Amazon Books (shuffled):For each book on Amazon presented in Fig. 1o we randomly redistributed the positive votes (4 and 5 stars) and the negative ones (1 and 2 stars), separately.
- Brazilian elections (state deputies in 2010):

At the 2010 electoral dispute, there were 27 eligible parties in Brazil: PMDB, PT, PP, PSDB, PDT, PTB, PTdoB, DEM, PR, PSB, PPS, PSC, PCdoB, PV, PRB, PRP, PMN, PSL, PTC, PSDC, PHS, PTN, PRTB, PSOL, PSTU, PCB, and PCO. We obtain the political orientation for each one of these parties from

http://en.wikipedia.org/wiki/List_of_political_parties_in_Brazil

(accessed on 11/22/2013):

- Extreme-left: PSTU, PCB, PCO (Total: 3)
- Left: PT, PSB, PCdoB, PSOL (Total: 4)
- Center-left: PSDB, PDT, PTB, PPS, PV, PMN (Total: 6)
- Center: PMDB, PTdoB, PRB, PRP, PSL, PHS, PTN, PRTB (Total: 8)
- Center-right: PTC, PSDC (Total: 2)
- Right: PP, DEM, PR, PSC (Total: 4)

We analyze the 2010 Brazilian election for state deputies, which correspond to state legislative assemblies representatives (Fig. 1q). These data are available at http://agencia.tse.jus.br/estatistica/sead/odsele/votacao_partido_munzona/votacao_partido_munzona_2010.zip.

For each city in Brazil, we compute the number of votes received by the parties associated to each one of the six political orientations. Note that there is no extreme-right party in Brazil. Arbitrarily, we take votes on extreme-left, left, and center-left parties as negative opinion, *N*^−^. The votes on the center, center-right, and right parties are considered as positive opinion, *N*^+^. We consider as extreme opinions the votes on extreme-left and left parties, 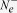, and the votes on center-right and right parties, 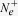, respectively. This choice is motivated by the fact that very small fractions of the electorate correspond to orientations of extreme-left and center-right. Currently, there are 32 parties in Brazil, where 5 new parties were created in the country since the 2010’s election. None of the parties in 2010 considered in the present work was dissolved.

## Author Contributions

H.A.M designed research, M.R, J.S., S.D.S.R, C.A., J.S.A. Jr., S.H. and H.A.M. performed research, and wrote the manuscript.

## Additional Information

**How to cite this article**: Ramos, M. *et al*. How does public opinion become extreme? *Sci. Rep.* **5**, 10032; doi: 10.1038/srep10032 (2015).
